# Palladium nanoparticles entrapped in a self-supporting nanoporous gold wire as sensitive dopamine biosensor

**DOI:** 10.1038/s41598-017-07909-y

**Published:** 2017-08-11

**Authors:** Xin Yi, Yuxuan Wu, Guoxin Tan, Peng Yu, Lei Zhou, Zhengnan Zhou, Junqi Chen, Zhengao Wang, Jinshan Pang, Chengyun Ning

**Affiliations:** 1 School of Medicine, South China University of Technology, Guangzhou, China; 2 School of Materials Science and Engineering, South China University of Technology, Guangzhou, China; 3 Institute of Chemical Engineering and Light Industry, Guangdong University of Technology, Guangzhou, China; 4 Department of Electronic Communication & Software Engineering, Nanfang College of Sun Yat-sen University, Guangzhou, China; 5 Guangdong Key Laboratory of Biomedical Sciences and Engineering, South China University of Technology, Guangzhou, 510006 China

## Abstract

Traced dopamine (DA) detection is critical for the early diagnosis and prevention of some diseases such as Parkinson’s, Alzheimer and schizophrenia. In this research, a novel self-supporting three dimensional (3D) bicontinuous nanoporous electrochemical biosensor was developed for the detection of dopamine by Differential Pulse Voltammetry (DPV). This biosensor was fabricated by electrodepositing palladium nanoparticles (Pd) onto self-supporting nanoporous gold (NPG) wire. Because of the synergistic effects of the excellent catalytic activity of Pd and novel structure of NPG wire, the palladium nanoparticles decorated NPG (Pd/NPG) biosensor possess tremendous superiority in the detection of DA. The Pd/NPG wire biosensor exhibited high sensitivity of 1.19 μA μΜ^−1^, broad detection range of 1–220 μM and low detection limit up to 1 μM. Besides, the proposed dopamine biosensor possessed good stability, reproducibility, reusability and selectivity. The response currents of detection in the fetal bovine serum were also close to the standard solutions. Therefore the Pd/NPG wire biosensor is promising to been used in clinic.

## Introduction

It is well-known that DA is an important catecholamine neurotransmtiter^1–3^. Abnormal level of DA will lead to some serious diseases such as Parkinson’s, Alzheimer and schizophrenia^4, 5^. The detection of DA is beneficial for early diagnosis and prevention. Therefore it is extremely necessary to develop DA biosensors with the brilliant performance such as high sensitivity, broad detection range and excellent selectivity, and so on. Recently, high performance liquid chromatography^6^, liquid chromatography-electrospray tandem mass spectrometry^7^, surface-enhanced Raman scattering spectroscopy and fluorescence^8^ detection methods were applied to detect DA. Although these methods possessed some fine property, they were poor in certain aspects of property, such as instrument complexity, low sensitivity and time consuming, and so on. Fortunately, the characteristics of electrochemical activity of DA make it detectable by electrochemical method^9^, which has attracted increasing attention, due to the advantages in sensitivity, detection time, selectivity and operation, and so on. However, the ascorbic acid (AA), uric acid (UA)^10^, norepinephrine (NE), epinephrine (EP)^11^ and catechol (CC)^12^ coexist with DA in a living organism and have some interferences during the detection of DA. Therefore, the electrochemical method used to detect DA must possess excellent selectivity on the basis of broad detection range, high sensitivity and low detection limit, and so on.

Enzyme electrochemical biosensors with the function of biorecognition had the advantage in selectivity for detection of biomolecule^13^. The enzyme electrochemical biosensors also possessed advantages such as broad detection range, high sensitivity, low detection limit, rapid sensing and ease in miniaturization, and can become a good choice for the detection of DA. However, the limitation of enzymes was affected by the environment easily^14, 15^, which seriously restricted the application of enzyme electrochemical biosensors. In order to solve this problem, researchers developed nonenzymatic electrochemical biosensors, which were promising to overcome the limitation of enzymes easily affected by the environment^16, 17^. The noble metal nanoparticles due to possessing some special property such as enhanced electrocatalytic activity^18^, excellent adsorption capacities^19^ and brilliant electron-transfer^20^, was a promising nonenzymatic catalysts^21^. Nevertheless, the inherent defect of nanoparticles aggregating easily^22^, prevented its application in sensing. For solving this problem, researchers attempted diverse methods, and supporting the noble metal nanoparticles by the solid supports obtained fascinating consequences^23–25^. It has reported that graphene^26^, and single-walled and multi-walled carbon nanotubes^27–29^ have been used to support the nanoparticles. These nanohybrids made the interface of electrode/electrolyte possess extraordinary large specific surface area^28^, but the nature of extremely poor charge transfer made these electrodes own an exceptionally poor electronic conductance weakening the sensing properties^30^. Besides, among the noble metal nanoparticles, conductive substance and current collector existed large contact resistances^31^, preventing their use in the detection area seriously. The low-dimensional nanostructure also weakened the superiority of these nanohybrids in sensing.

In order to resolve this problem, we constructed a nanoporous structure, which not only prevent the aggregating of nanoparticles, but also can take advantage of nanostructure. Lang *et al*. have reported that NPG supported cobalt oxide microelectrodes possess excellent performance in the detection of glucose^32^. Han *et al*. have reported that self-grown Ni(OH)_2_ layer on bimodal nanoporous AuNi alloys can enhanced electrocatalytic activity^33^. In this work, we report a unique self-supporting three dimensional (3D) NPG with self-supporting bicontinuous nanoporosity. In this structure, the NPG wire not only possesses brilliant conductivity, accelerating charge transfer^34, 35^, but also can support itself. Besides, the NPG wire also own bicontinuous nanoporous structure, which is beneficial for the mass transport^36^. In order to improve the sensing performance for DA further, we inserted Pd into the NPG wire, due to the Pd owning admirable electrocatalytic for DA^37, 38^, and fabricated the Pd/NPG wire DA biosensor. To the best of our knowledge, there is no report about the Pd/NPG wire biosensor used to detect DA. The synergistic effects of the excellent catalytic activity of Pd and novel structure of NPG wire make the Pd/NPG wire biosensor possess tremendous superiority in the detection of DA. Also, the direct connection method decreased the contact resistances among Pd, nanoporous Au skeleton and current collector of solid Au wire at the same time, improving the sensing property. The unique structure of interconnected nanoporous channels and ligaments, not only offers fast DA transfer, but also enlarges the specific surface area of electrode/electrolyte interface, improving the performance of the Pd/NPG wire biosensor. All discussed above make the Pd/NPG wire biosensor own excellent sensing performance.

## Results and Discussion

### Characterization of the Pd/NPG wire biosensor

**Figure 1:**
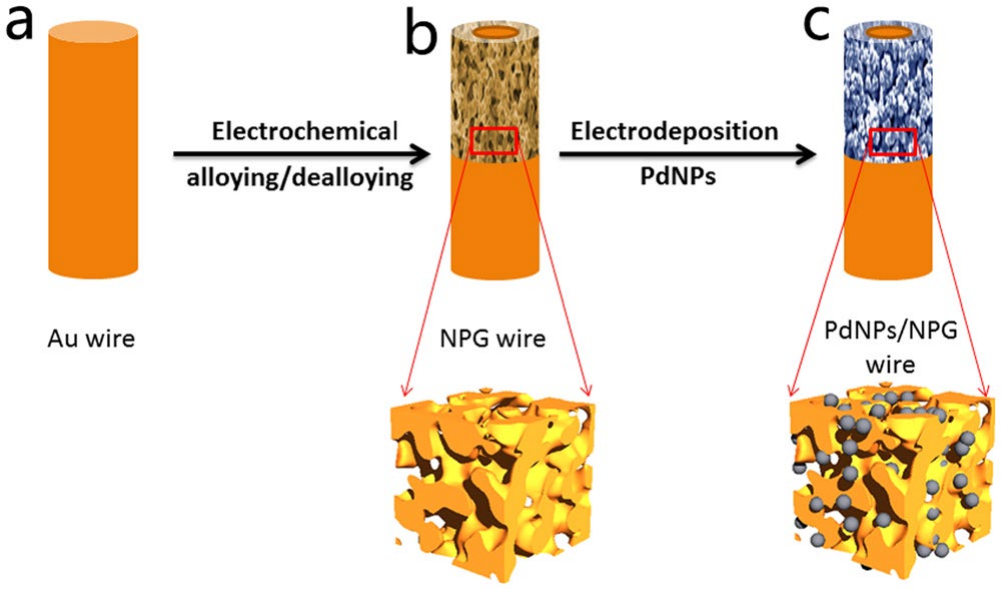
Schematic diagram for the fabrication of the biosensor. (**a**) Au wire. (**b**) 3D bicontinuous nanoporous gold wire fabricated by electrochemical alloying/dealloying. (**c**) Pd decorated NPG wire.

Our strategy to improve the sensing performance of DA is to fabricate Pd/NPG wire biosensor with the interconnected nanoporous structure. Figure 1 shows the synthesis of Pd/NPG wire biosensor. Firstly, the NPG wire with the 3D bicontinuous nanoporous structure was fabricated by electrochemical alloying/dealloying. Secondly, Pd was electrodeposited onto NPG wire to improve the electrocatalytic activity for DA.

**Figure 2:**
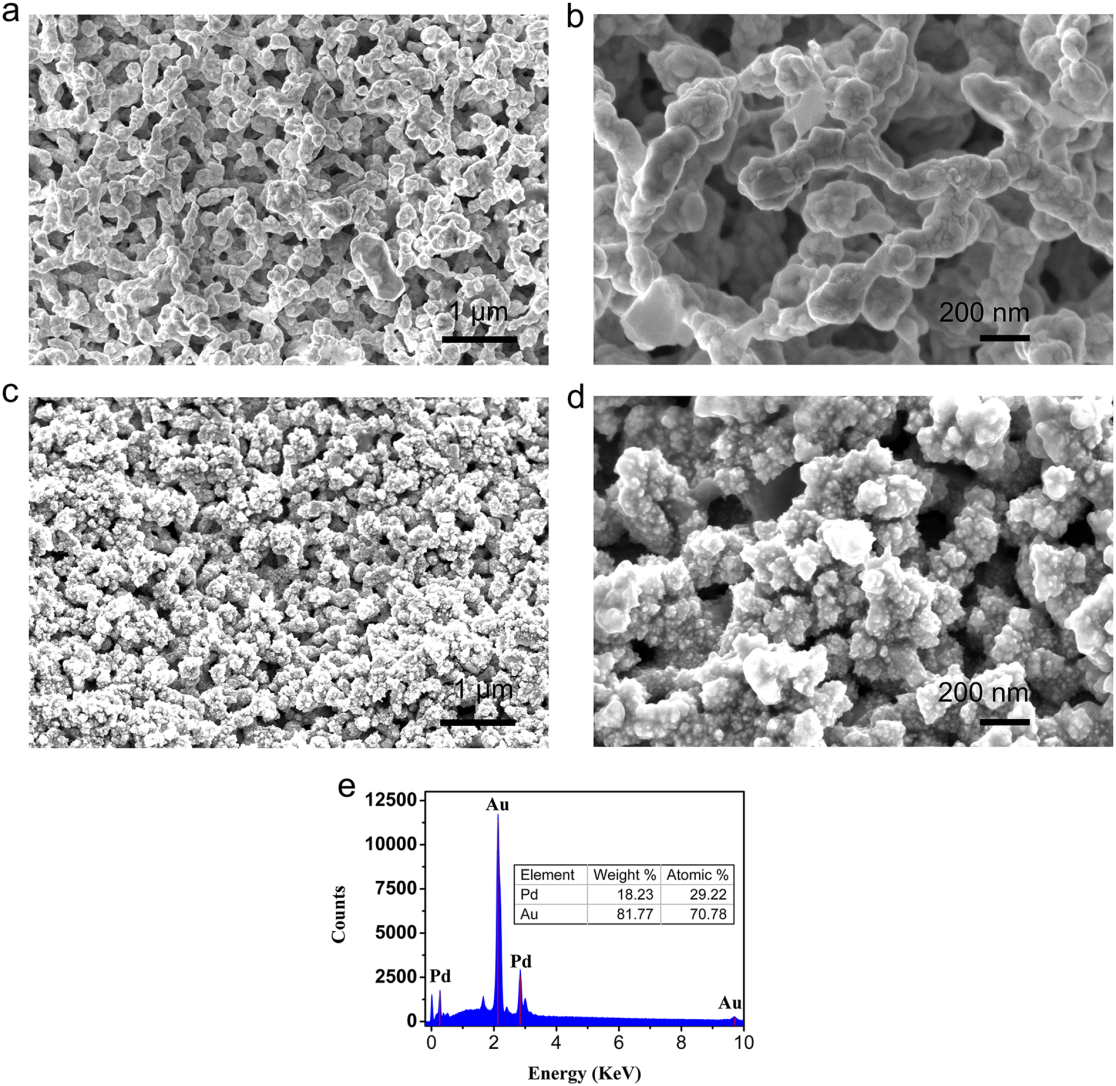
Microstructure characterization of the biosensor. (**a**) Low-magnification and (**b**) high-magnification SEM images of NPG wire fabricated by electrochemical alloying/dealloying, showing the 3D bicontinuous nanoporous structure. (**c**) Low-magnification and (**d**) high-magnification SEM images of Pd/NPG wire prepared by Pd electrodepositing on NPG wire, indicating the Pd distributing along the ligament of NPG wire uniformly. (**e**) Energy dispersive spectrum of Pd/NPG wire.

Figure 2a shows the surface topography of NPG wire fabricated from the smooth Au wire with a diameter of 200 μm, and the NPG wire was composed of Au ligaments and connected nanopores (Fig. S1). The SEM images indicated that the NPG wire possessed uniform 3D bicontinuous nanoporous structure and the ligaments of NPG with the characteristic length of ~200 nm (Fig. 2b). This unique architecture possesses large specific surface area which is beneficial for the load of the Pd.

Figure 2c provides the SEM image of Pd/NPG wire, indicating the 3D bicontinuous nanoporous structure still exists after decorated by Pd, and this structure is advantageous for the transport of DA and electron. As Fig. 2d shown, the Pd distributed along with the ligaments of NPG wire uniformly and directly, and possessed the characteristic length of ~20 nm (Fig. S1d). Figure S2 showed the distributing of Pd. The red color represents the distributing of Pd, and the black color represents the nanoporous channels of NPG. From the Fig. S2, we can know that the Pd distributed along with the ligaments of NPG wire uniformly. The direct connection avoided the additional contact resistance between Pd and NPG wire, improving the performance of the biosensor. The incorporation of Pd with the diameter of 10 nm (Fig. S1d) further enlarges the interface of the electrode and electrolyte, which is advantage for the oxidation of DA. Figure 2e shows the EDS spectrum of the Pd/NPG wire, indicating the existence of Pd and Au from the corresponding peaks of two elements. The percentage of Pd and Au is can be obtained from Fig. 2e, the percentage of weight is 18.23% and 81.77% respectively, and the percentage of atomic is 29.22% and 70.78% respectively. This method makes the electrode own additional superiority such as large specific surface area, fast transports of DA and excellent electrocatalytic for DA, on the basis of brilliant electrical conductivity of bulk Au, improving the sensing property of the biosensor.

### Electrochemical features of the Pd/NPG wire biosensor

**Figure 3:**
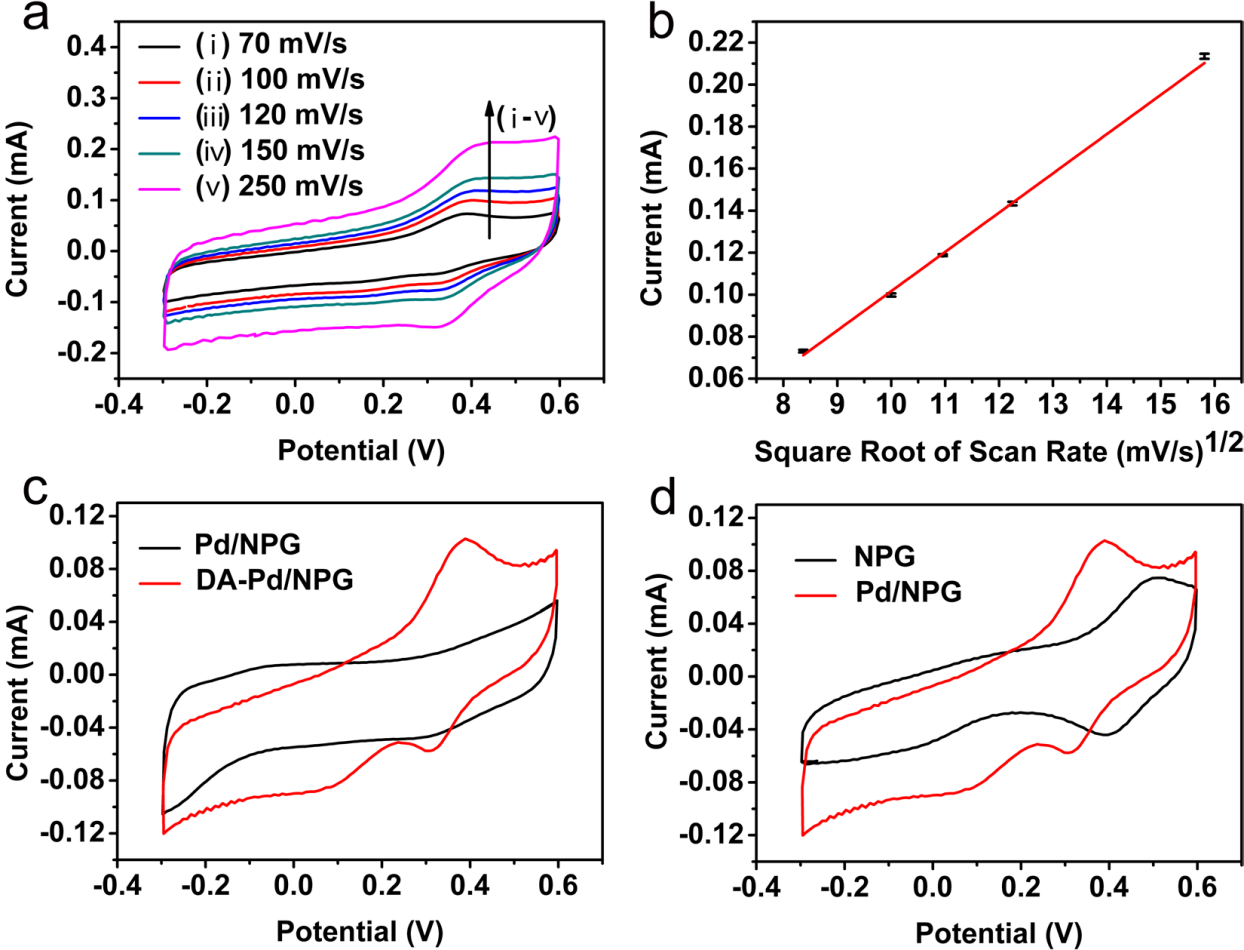
Electrochemical characterazations of the Pd/NPG wire biosensor. (**a**) The CV curves of the Pd/NPG wire biosensor at various scan rates (from (i) to (v): 70, 100, 120, 150 and 250 mV/s). (**b**) The calibration plot of the oxidation peak current of DA versus the square root of scan rate (70–250 mV/s). (**c**) The CV curves of Pd/NPG wire biosensor in the PBS containing 0 μM and 100 μΜ DA at the scan rate of 100 mV/s. (**d**) The CV curves of NPG wire biosensor and Pd/NPG wire biosensor in the PBS containing 100 μM DA at the scan rate of 100 mV/s.

The Fig. 3a shows the CV curves of the Pd/NPG wire biosensor with varying scan rate from 70 to 250 mV/s in the solution of phosphate buffered saline (PBS) containing 100 μΜ DA to research the performance of the electrode. It is obvious that the anodic peak value of DA become more positive potentials along with the increase of scan rate. This indicates that there are maybe electropolymerization of DA and it is more distinct at higher scan rate, and show that a low scan rate, for example 100 mV/s, is suitable for the detection of DA. The anodic peak current (I_pa_) of DA increases with the square root of scan rate linearly (Fig. 3b). The function about I_pa_ of DA versus the square root of scan rate obtained from Fig. 3b is

1 $${I}_{pa}=18.63\times {(scanrate)}^{1/2}-84.26\,({R}^{2}=0.998),$$

which indicates that the oxidation of DA was controlled by the diffusion of DA. The surface concentration of ionic species of the NPG wire and Pd/NPG wire electrodes can get from Brown–Anson model (equation. (2))^39^.

2 $${I}_{p}={n}^{2}{F}^{2}Av/4RT$$

where I_p_ represent the peak current, n represent the number of electrons transferred (n = 1), F represent the Faraday constant (F = 96485 C/mol), γ represent the surface concentration of ionic species on NPG wire and Pd/NPG wire electrodes (mol/cm^2^), A represent the surface area of the electrode (A = 0.1 cm^2^), v represent the scan rate (v = 100 mV/s), R represent the gas constant (R = 8.314 J/mol·k) and T represent the room temperature (T = 25 °C). It is noted that the surface concentration of ionic species on Pd/NPG wire electrode (3.16 × 10^−9^ mol/cm^2^) is higher as compared to the NPG wire electrode (2.27 × 10^−9^ mol/cm^2^). The diffusion co-efficient value (D) of electrolyte solution to the NPG wire and Pd/NPG wire electrode surface can be got from Randles–Sevcik equation (equation. (3))^39^.

3 $${I}_{p}=(2.69\times {10}^{5}){n}^{3/2}A{D}^{1/2}C{v}^{1/2})$$

where, D represent the diffusion co-efficient and C represent the surface concentration in mol (C = 5 mM). The diffusion co-efficient value of Pd/NPG wire electrode (4.85 × 10^−7^ cm^2^/s) is higher than that of NPG electrode (2.57 × 10^−7^ cm^2^/s). The electroactive surface area (A_e_) of Pd/NPG wire electrode has been obtained using Randles–Sevcik equation (equation. (4)) and calculated diffusion co-efficient value.

4 $${A}_{e}=S/(2.99\times {10}^{5}){n}^{3/2}C{D}^{1/2}$$

where, S represents the slope of straight line obtained from equation. (1). The A_e_ value for Pd/NPG electrode has been estimated as 34.38 mm^2^.

To confirm the occurrence of DA redox reaction on Pd/NPG wire biosensor, the study was performed in the PBS with 0 μM and 100 μM DA by the method of CV on Pd/NPG wire biosensor. As shown in Fig. 3c, the curve of Pd/NPG wire biosensor show there is no definite anodic peak in the electrolyte without DA, indicating there is not the redox reaction occurrence of DA. However, in the electrolyte containing 100 μM DA, an obvious anodic peak of DA appears at 0.4 V, which represents the DA oxidized to dopamine quinone. And this anodic peak of 0.4 V is lower than others report, such as Zhao *et al*. (0.45 V) and Roychoudhury (1.1 V), which is that Pd/NPG wire biosensor has more high catalytic activity than that Besides, there are two cathodic peak in 0.3 V and 0.1 V, which are dopamine quinone reduced to DA and 5,6-dihydroxylindole reduced to 5,6-indolequinone respectively (dopamine quinone can form 5,6-dihydroxylindole during the electropolymerization)^40^. By the comparison of the response current, it demonstrates the anodic peak appearing at 0.4 V is due to the oxidation of DA existing in electrolyte.

To confirm the Pd/NPG wire biosensor owning higher catalytic activity for DA than NPG wire biosensor, NPG wire biosensor and Pd/NPG wire biosensor were used to detect 100 μΜ, 50 μΜ and 150 μΜ DA in the PBS. As shown in Figs 3d and S3, The oxidation peak of DA on the Pd/NPG wire biosensor is higher than that obtained from the NPG wire biosensor, and the onset potential of the Pd/NPG wire biosensor is more negative than that of NPG wire biosensor obviously. These is because of the Pd/NPG wire biosensor owning higher electrocatalytic activities than the NPG wire biosensor, and the higher electrocatalytic activities is obtained from the excellent electrocatalytic of Pd to DA. Therefore, inserting Pd into the NPG wire enhances the detection ability for DA dramatically. Also, the higher current can prove the Pd electrodeposited on the NPG wire successfully. In order to study the effect of the Pd loading on the oxidation of DA, we have synthesized samples with different Pd loading. As shown Fig. S4f, the curve of i, ii, iii, iv and v represent the CV curve of samples with different Pd loading, which was fabricated by CV after five, six, seven, eight and nine cyclic and the oxidation peak current of iii is higher than others, and it owns the best electrochemical performance comparing with others. Along with the increase of Pd (Fig. S4a–e), the active site of DA is increase, which is benefit for the oxidation of DA. However, when the load of Pd is too big, it will decrease the space of channel of the NPG, which will hinder the transport of DA and the oxidation of DA.

The interface characteristic of electrode and electrolyte is related to the property of the biosensor, and it was detected by EIS. Figure S5 provides the nyquist diagrams of electrochemical impedance spectra of the NPG wire biosensor and the Pd/NPG wire biosensor. The semicircular segment at higher frequencies represents the charge transfer resistance (R_ct_) and the linear part at lower frequencies represents the diffusion process. As shown in Fig. S5, the Pd/NPG wire biosensor possesses the smaller semicircle diameter of the EIS, compared to the EIS of the NPG wire biosensor, indicating the decreased resistance. The incorporation of Pd decreases the impedance of the NPG wire biosensor. It demonstrates the formation of Pd/NPG wire biosensor again.

### Electrochemical analysis of DA on Pd/NPG wire biosensor

**Figure 4:**
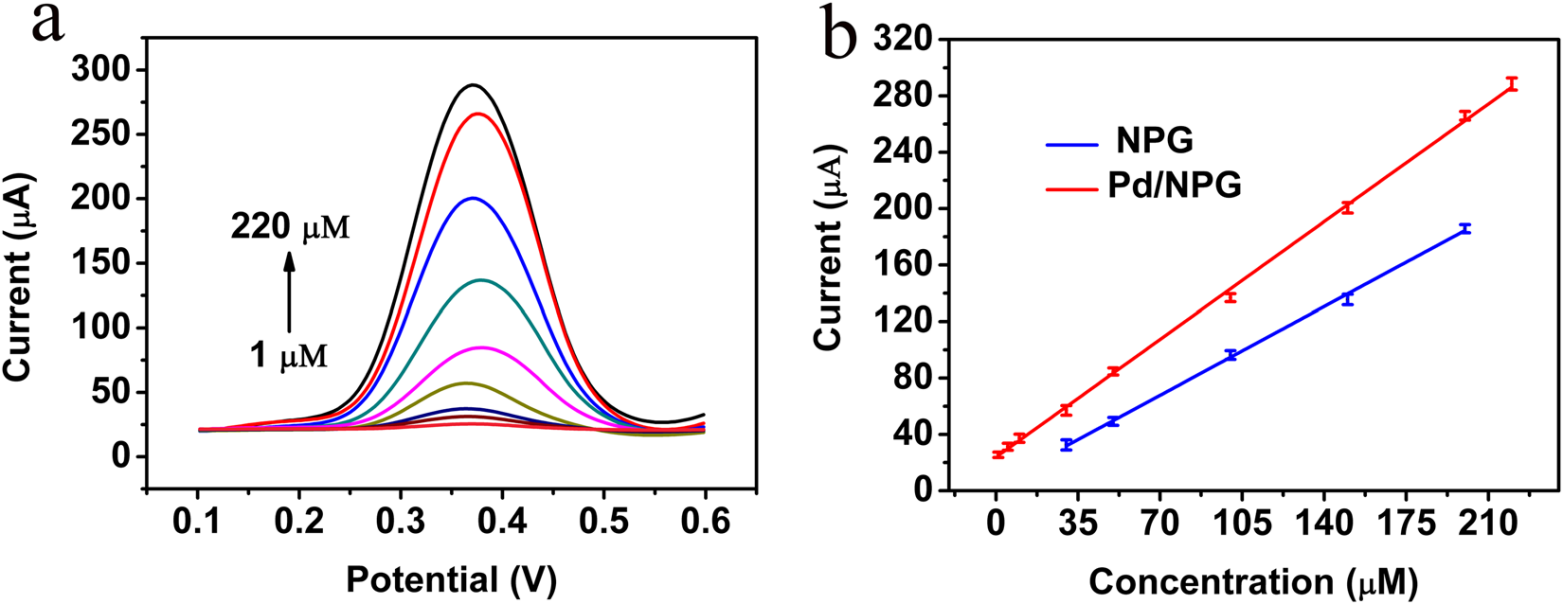
Detection of dopamine. (**a**) DPV curves of the Pd/NPG wire biosensor at various concentrations of DA (from 1 to 220 μΜ). (**b**) Plots of the anodic peak currents versus the concentrations of DA of Pd/NPG wire biosensor (1, 5, 10, 30, 50, 100, 150, 200, 220 μΜ) and NPG wire biosensor (30, 50, 100, 150, 200 μΜ).

To estimate the detection property of Pd/NPG wire biosensor for DA, the detection tests are performed by DPV. Figure 4a shows the DPV curves of various concentrations of DA in the electrolyte of PBS. The anodic peak current of DPV increases along with the change of concentration from 1 to 220 μΜ, which is ascribed to the oxidation of more DA. The more detailed information of this change can learn from the calibration plot of Pd/NPG wire biosensor (Fig. 4b), and the linearity relationship is

5 $${I}_{pa}=1.19\times concentration\,+23.32\,({R}^{2}=0.996).$$

The sensitivity of the Pd/NPG wire biosensor can obtain from the slope of calibration plot and it is 1.19 μA μΜ^−1^. Also, the detection range and the detection limit of the Pd/NPG wire biosensor is 1–220 μM and 1 μM respectively. For comparison, the calibration plot of NPG wire biosensor also was shown in Fig. 4b, the detection range is from 30 to 200 μΜ, and the detection range of Pd/NPG wire biosensor is much wider than NPG wire biosensor. Owing to the incorporation of Pd, Pd/NPG wire biosensor possesses higher catalytic activity than NPG wire biosensor for DA, and exhibits wider detection range. Besides, the current-time curves shown that the Pd/NPG wire biosensor owned excellent stability and short response time (Fig. S6).

The superiority of this biosensor to others can be showed by the comparison with others (Table S1), in the range, sensitivity and limit of the detection. The sensitivity (1.19 μA μΜ^−1^) and the detection range (1–220 μΜ) of this work is superior to other reported such as Hu *et al*. (0.33 μA μΜ^−1^ and 1–6 μΜ), Bao *et al*. (0.27 μA μΜ^−1^ and 1–14 μΜ), Sheng *et al*. (0.03 μA μΜ^−1^ and 0.5–170 μΜ), Roychoudhury *et al*. (0.06 μA μΜ^−1^ and 2–100 μΜ), Yang *et al*. (0.48 μA μΜ^−1^ and 0.5–60 μΜ) and Yang *et al*. (1.04 μA μΜ^−1^ and 1–50 μΜ). Although the sensitivity of Yang *et al*. reported the sensitivity (2.31 μA μΜ^−1^) is higher than this work, the detection range (1–50 μΜ) is poor. The comparison reveals the high comprehensive property of the Pd/NPG wire biosensor, and proves the potentials application in the detection of DA.

**Figure 5:**
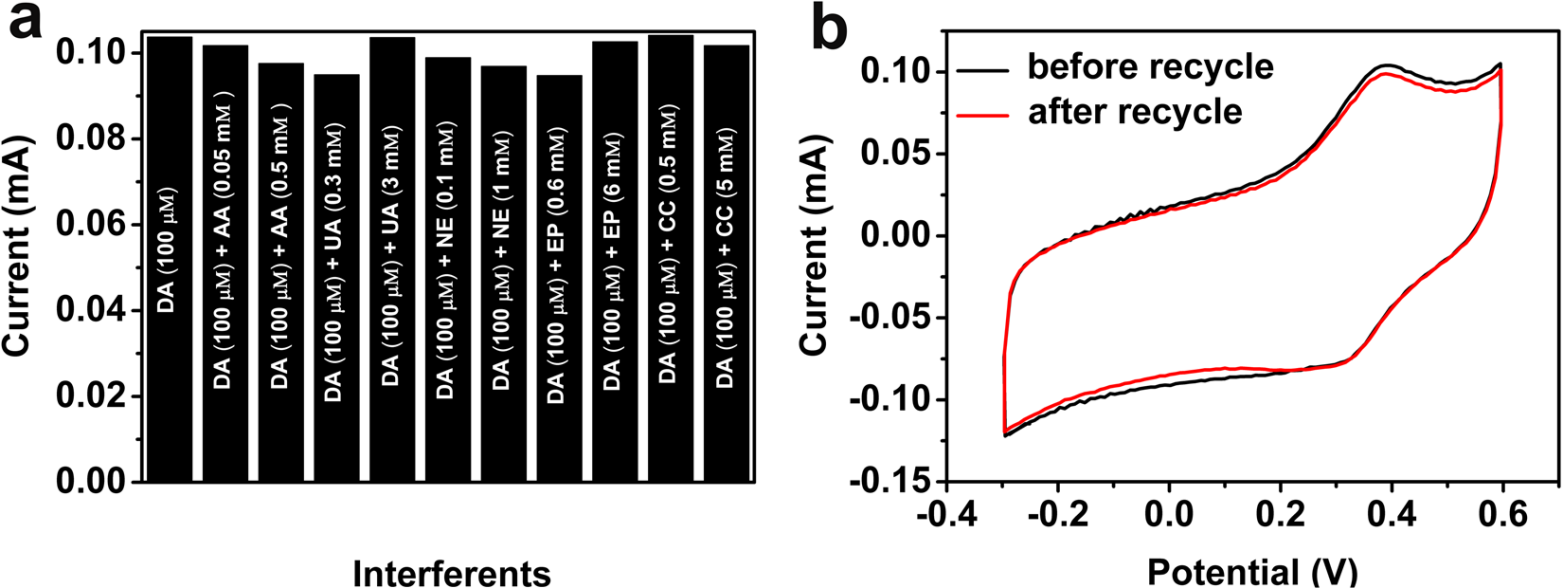
Specificity and reusability of the Pd/NPG wire biosensor. (**a**) Response currents of the Pd/NPG wire biosensor in the solution of 100 μΜ DA in the presence of AA, UA, NE, EP and CC. (**b**) The CV curves of Pd/NPG wire biosensor before and after recycle.

The specificity of the Pd/NPG wire biosensor was studied by detection the DA with the concentration of 100 μΜ, in the presence of AA, UA, NE, EP and CC which are co-existed with DA in physiological fluids^10^. As shown of Fig. 5a and Fig. S7, the response currents did not change obviously, and it was less than 9% (RSD value ~3–10%) (Table S2), with the interferences of AA (0.05 mM and 0.5 mM), UA (0.3 mM and 3 mM), NE (0.1 mM and 1 mM), EP (0.6 mM and 6 mM) and CC (0.5 mM and 5 mM)). This own to the efficient catalysis of Pd to DA at 0.4 V. The high selectivity makes this biosensor own the excellent reliability for detection of DA in blood serums.

Considering the cost of gold, the repeated use of NPG wire form the composite of Pd/NPG wire biosensor is a feasible idea. The NPG wire can be obtained by immersing the composite of Pd/NPG wire biosensor into the concentrated HNO_3_ for 2 hour. The CV curves of Fig. 5b, shows the peak currents are 0.1029 mA and 0.09894 mA respectively, and that the response current retained 96.15% after recycle comparing with before recycle. Figure S8 shows the microstructure feature of NPG wire and Pd/NPG wire after recycle, and the 3D bicontinuous nanoporous structure still exist, which also proves the superior reusability. The excellent reusability may be come from the good stability of NPG wire.

**Figure 6:**
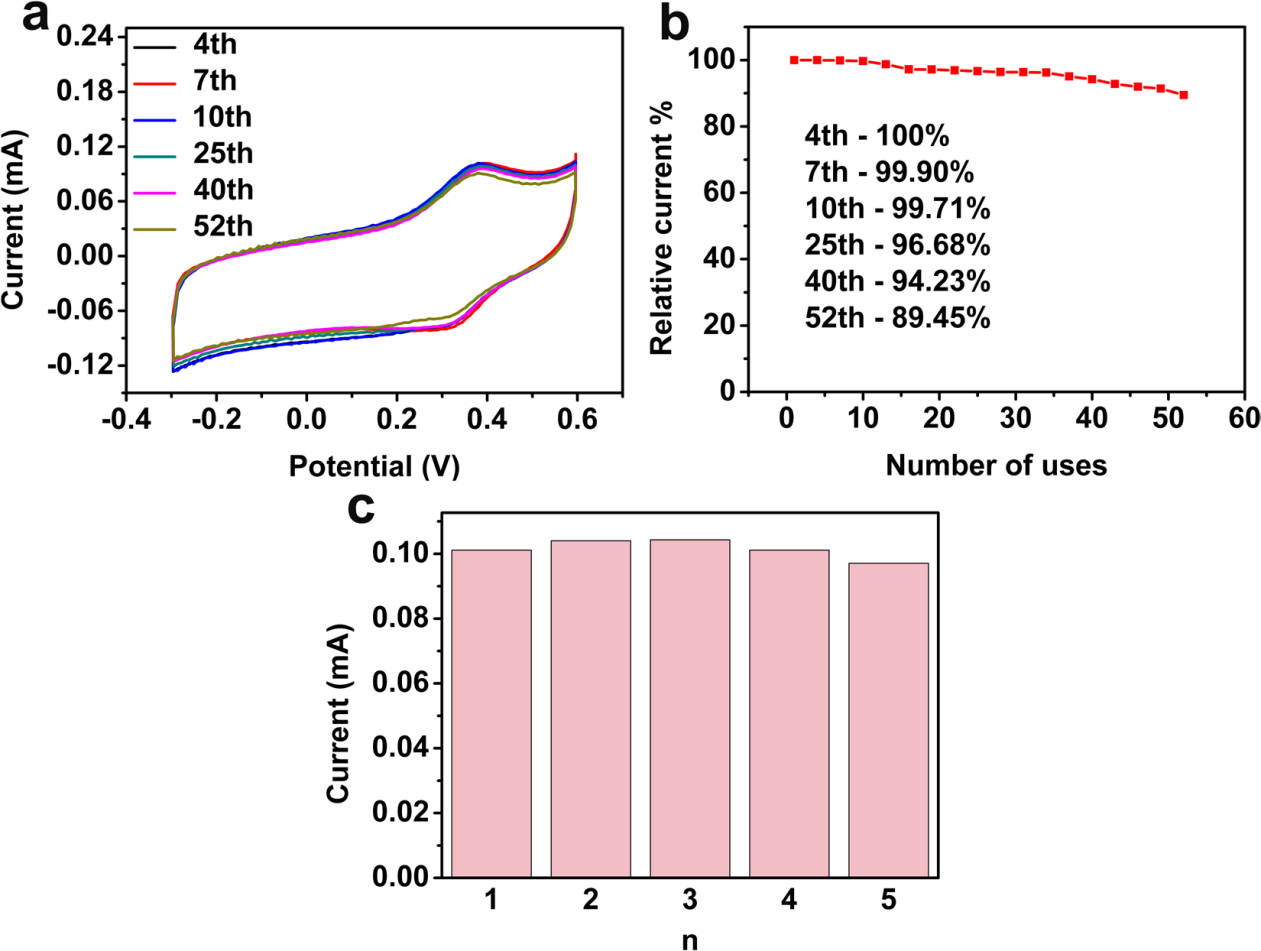
Stability and reproducibility of the Pd/NPG wire biosensor. (**a**) Response currents of the Pd/NPG wire biosensor under different measurements. (**b**) Stability study of the Pd/NPG wire biosensor. (**c**) Reproducibility study on Pd/NPG wire biosensor.

The stability (Fig. 6a) of Pd/NPG wire biosensor was studied in the PBS containing 100 μΜ DA. The Fig. 6b shows CV curves of the 4th, 7th, 10th, 25th, 40th, 52th measurements. Figure 6b shows the response current of the 4th measurements still retain 100%, and the 40th measurements retain 94.23%, compared to the first time, which proves this biosensor of DA owning excellent stability at the first 40 times. However, the 52th not only has obvious decrease, but also has a clear shift of the dopamine oxidation process towards more positive potentials. This is because of dopamine being electropolymerized on the surface of the electrode, and shows that the use time should not over 40 times. Besides, the RSD is 9.74% of the 40th measurements. The solid support preventing the aggregation of Pd, may be the primary reason for brilliant stability of this biosensor. In order to ensure the reliability of the biosensor, the property of reproducibility is also need to examine. The reproducibility of this biosensor was studied by five biosensors fabricated at the same condition to test in the solution containing 100 μΜ DA. As show of Fig. 6c and Fig. S9, the relative standard deviation (RSD) of results was 2.81%, indicating this biosensor possessing a fascinating reproducibility. The fascinating reproducibility is come from the superiority of electrochemical alloying/dealloying and electrochemical deposition.

In order to demonstrate interference of other electrochemical active substances existing in biological fluids during the detection of DA, and estimate the performance of Pd/NPG wire biosensor in the application of diagnosis and prevention, the assay of DA in fetal bovine serum samples is necessary. Briefly, diverse concentration of DA (10, 20, 30, 40, 50 μΜ) were added into the fetal bovine serum samples diluted 15 times by PBS (pH 7), comparing the response current with the response current of standard samples. The Table 1 shows that the results of the detection in fetal bovine serum samples and standard samples have a good accordance, and the variation range of recoveries was 97.3% to 103.8%. This result shows the Pd/NPG wire biosensor has a good anti-interference performance for the electrochemical activity substance, for example protein existing in biological fluids. The protein content which exist in fetal bovine serum sample is much large than in body fluids, so Pd/NPG wire biosensor is promising to been used in the detection of DA in the area of diagnosis and prevention.

**Table 1:** Serum sample analysis and the comparison with standard samples (n = 5).

Number	Labeled (μΜ)	Detected (μΜ)	Recovery (%)	RDS (%)
1	10.00	9.81±	98.10	4.61
2	20.00	19.49±	97.50	3.81
3	30.00	30.37±	101.20	3.52
4	40.00	41.53±	103.80	5.42
5	50.00	48.67±	97.30	4.76

## Conclusion

A novel nonenzymatic electrochemical biosensor based on the Pd/NPG wire was fabricated for the detection of DA. The synergistic effects of the excellent electrocatalysis of Pd for DA and the 3D self-supporting bicontinuous nanoporous structure of NPG wire greatly enhance the response current and improve the electrochemical signal. Therefore, this biosensor possessing high sensitivity, broad detection range and excellent selectivity is promising for applications of DA in diagnosis and prevention.

## Experimental section

### Materials and reagents

Gold wires with the diameter of 200 μm (purity of 99.99%) were purchased from Changshu Changhong Precious Metal Co., Ltd. (Jiangsu, China). Palladium dichloride (PdCl_2_), benzyl alcohol (BA), zinc chloride (ZnCl_2_), sodium dodecyl sulfate (SDS), DA, AA, UA, NE, EP, CC, potassium ferricyanide (K_3_Fe(CN)_6_), potassium ferrocyanide (K_4_Fe(CN)_6_), and potassium chloride (KCl) were purchased from Aladdin Reagent Company (Shanghai, China). Phosphate buffered saline was purchased from Shanghai Double-Helix Biotech co., Ltd. All other chemical reagents were analytical reagents grade and directly used without further purification. The length of all used gold wire is 1.5 cm, and the surface area is 0.09 cm^2^.

### Preparation of NPG wire

The NPG wire were fabricated by the method of electrochemical alloying/dealloying, with a three electrode electrochemical system at the temperature of 120 °C in ZnCl_2_ solution^41^. Firstly, the alloy of Zn-Au was formed by electrochemical alloying. Then, the Zn atom was dissolved by electrochemical dealloying, and the Au atom without any loss. The auxiliary electrode, the reference electrode and the working electrode were Zn plate, Zn wire and gold wire with diameters of 200 μm respectively. The electrolyte was prepared by heating the mixed solution of BA containing 1.5 M ZnCl_2_ to 80 °C stirring for several hours. The potential range used in the working electrode was from −0.72 to 1.8 V (vs. Zn), under the scan rate of 7 mV·s^–1^. After fifty cyclic, the NPG wire was fabricated with appropriate characteristic length.

### Preparation of Pd/NPG wire

Pd was electrodeposited onto NPG wire according to certain literature methods^42^. Briefly, Pd/NPG wire was formed by electrochemical deposition, in the mixed solution of 5 mM SDS and 2.5 mM palladium chloride. The auxiliary electrode, the reference electrode and the working electrode were platinum wire, saturated calomel electrode and NPG wire respectively. The potential range applied to the working electrode was from 0.2 to 1.2 V (vs. SCE) at the scanning rate of 20 mV s^−1^. After certain cyclic, the Pd was deposited on the ligament of the NPG wire with appropriate thickness.

### Instruments and measurements

The scanning electron microscope (SEM) images and energy dispersive x-ray spectra (EDS) were obtained from a field emission scanning electron microscopy (FE-SEM, ZEISS Ultra 55, Germany). All electrochemistry measurements were carried out on an electrochemical workstation (Zennium Zahner Germany), with a three-electrode system: modified NPG wire electrode as the working electrode, a saturated calomel electrode (SCE) as the reference electrode, and a platinum wire electrode as the counter electrode. CV and DPV were performed in the electrolyte of phosphate buffered saline. Real sample analysis was performed in the solution of fetal bovine serum. The specificity of the biosensor was studied with the presence of AA, UA, NE, EP and CC. EIS was performed in the electrolyte of 5.0 mM [Fe(CN)_6_]^3−/4−^. All measurements were performed at room temperature.

## Electronic supplementary material

Supplementary Information
