# Supplementary material for: Palladium nanoparticles entrapped in a self-supporting nanoporous gold wire as sensitive dopamine biosensor
Source: Sci Rep. 2017 Aug 11;7:7941. doi: 10.1038/s41598-017-07909-y (PMC5554298; doi:10.1038/s41598-017-07909-y)
Supplement: Supplementary file 1 — Supplementary Information [file 41598_2017_7909_MOESM1_ESM.docx]

**Palladium nanoparticles entrapped in a self-supporting nanoporous gold wire as sensitive dopamine biosensor**

Xin Yi^a,e^, Yuxuan Wu^d^, Guoxin Tan^c^, Peng Yu^b,e*^, Lei Zhou^b,e^, Zhengnan Zhou^b,e^, Junqi Chen^b,e^, Zhengao Wang^b,e^, Jinshan Pang^b,e^, Chengyun Ning^b,e*^

^a^School of Medicine, South China University of Technology, Guangzhou, China

^b^School of Materials Science and Engineering, South China University of Technology, Guangzhou, China

^c^Institute of Chemical Engineering and Light Industry, Guangdong University of Technology, Guangzhou, China

^d^Department of Electronic Communication & Software Engineering, Nanfang College of Sun Yat-sen University, Guangzhou, China

^e^Guangdong Key Laboratory of Biomedical Sciences and Engineering, South China University of Technology, Guangzhou 510006, China

^*^Corresponding Author.

E-mail addresses: conan424683@live.com (P Yu), [imcyning@scut.edu.cn](mailto:imcyning@scut.edu.cn) (Prof. CY Ning).


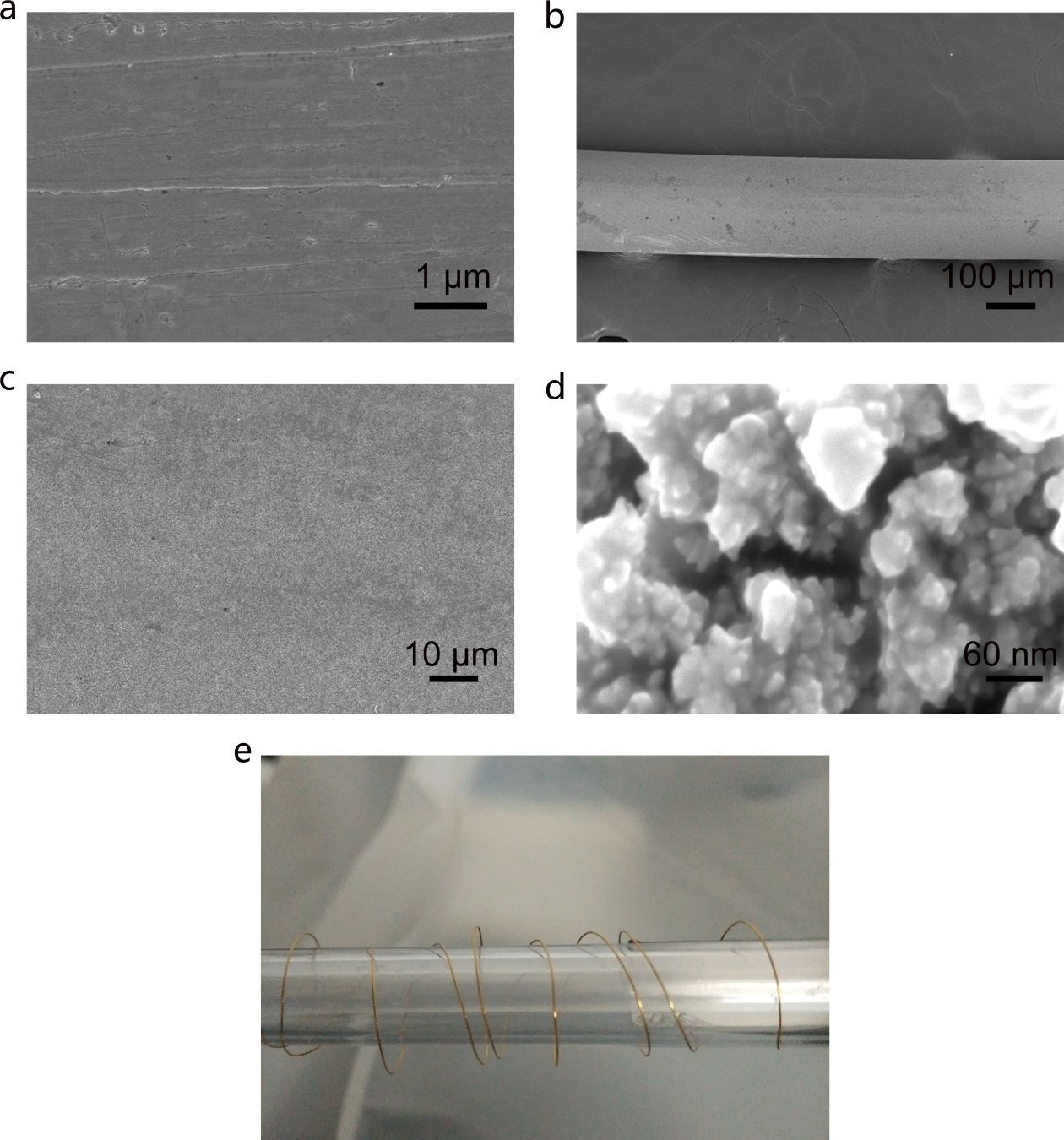


**Fig. S1.** (a) SEM image of a gold wire. (b), (c) and (d) SEM image of Pd/NPG. (e) Photograph of a gold wire.


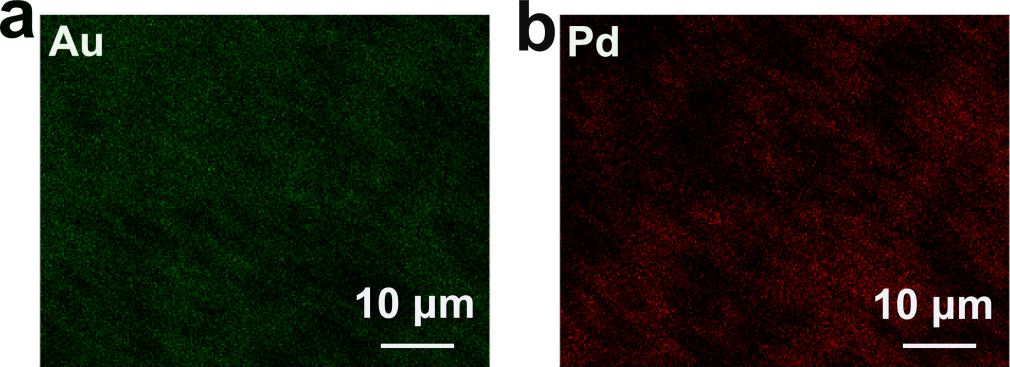


**Fig. S2.** EDS maps of the Pd and Au. (a) the map of Au, (b) the map of Pd.


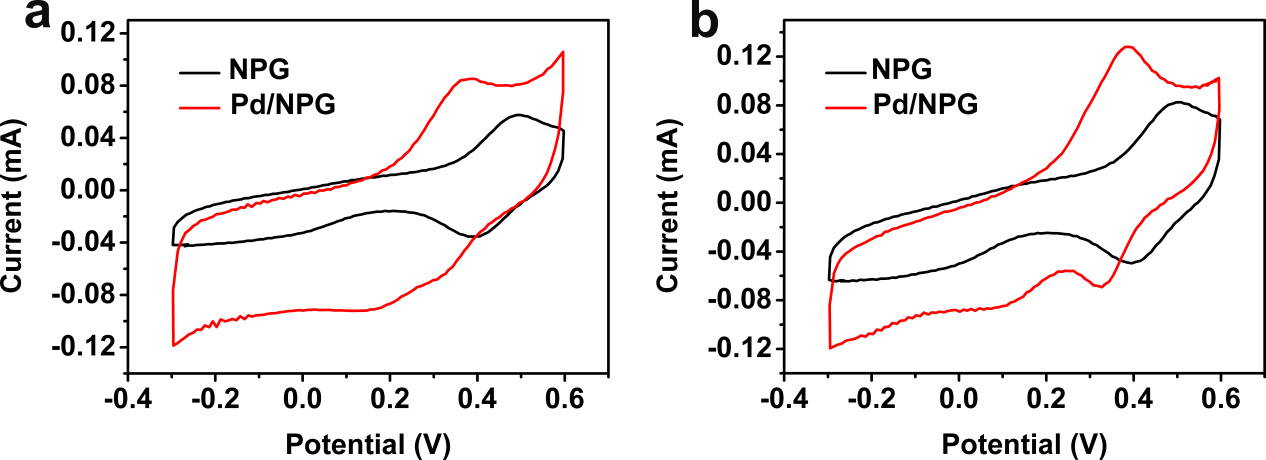


**Fig. S3.** CV curves of NPG wire biosensor and Pd/NPG wire biosensor in the PBS containing (a) 50 μM and (b) 150 μM DA at the scan rate of 100 mV/s.


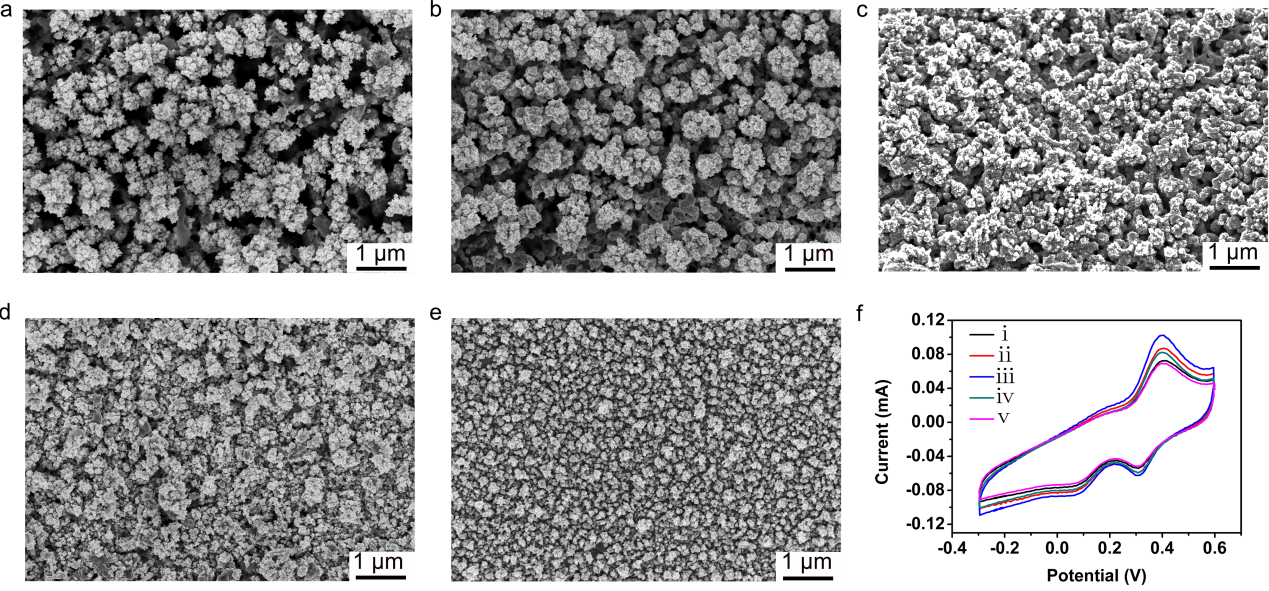


Fig. S4. (a-e) The SEM of Pd/NPG wire biosensors with different loading of Pd. (f) The CV of Pd/NPG biosensors with different loading of Pd (the curve of ⅰ, ⅱ, ⅲ, ⅳ and ⅴ represent the CV curve of samples with different Pd loading, which was fabricated by CV after five, six, seven, eight and nine cyclic).


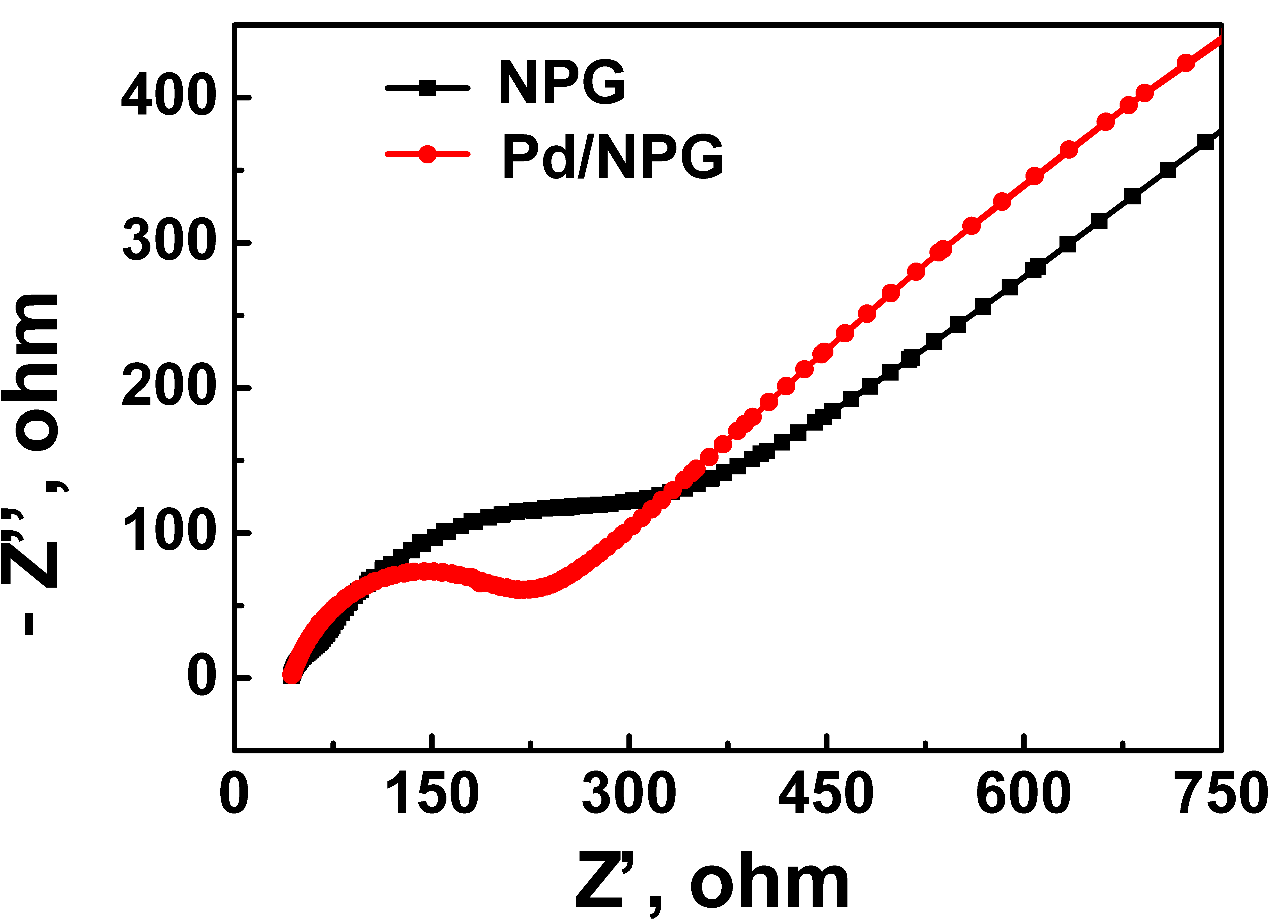


**Fig. S5.** The EIS of NPG biosensor and Pd/NPG biosensor.


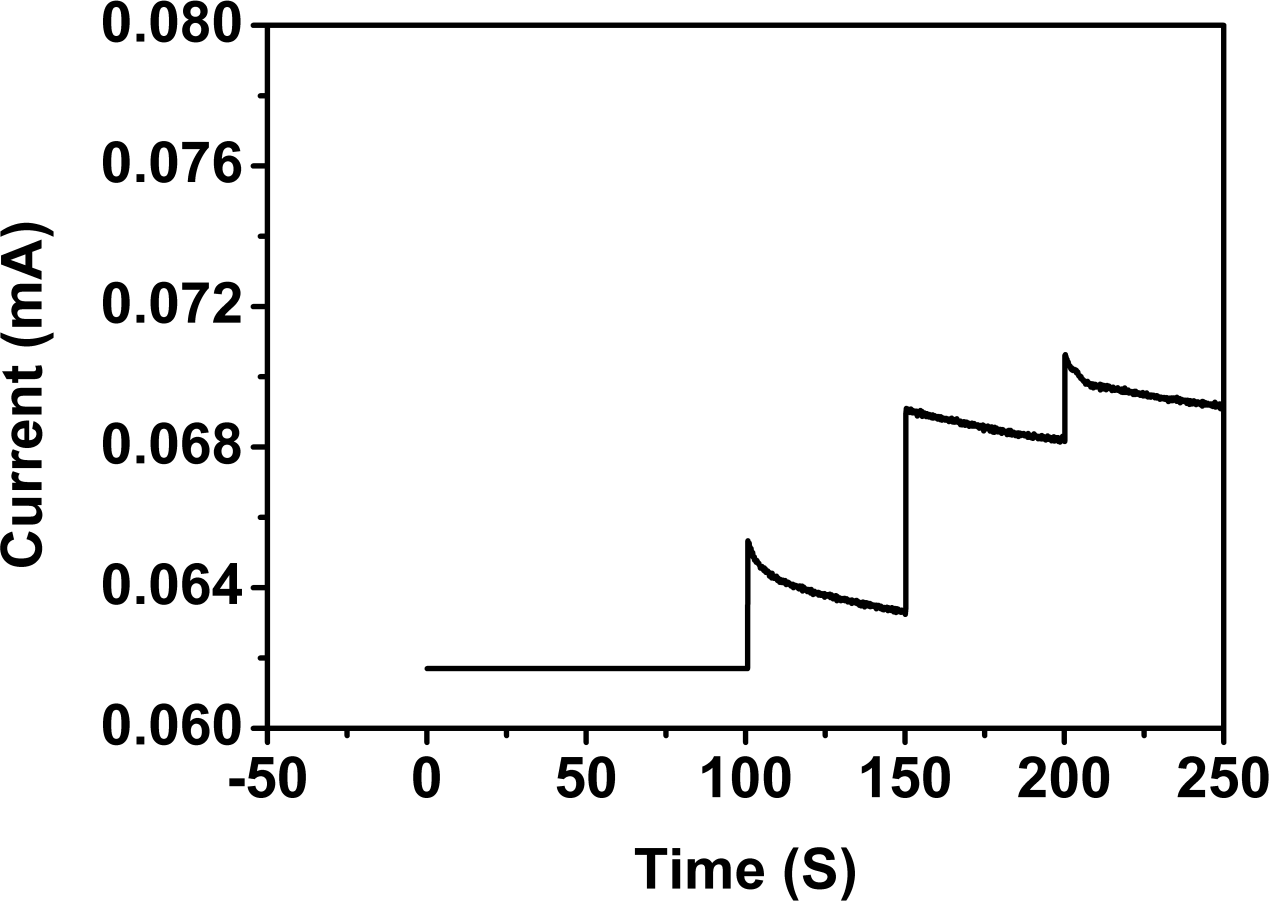


**Fig. S6.** Chronoamperometry curves for the amperometric responses of seamless Pd/NPG biosensor to different concentrations of DA (0, 1, 3, 5 μM) in PBS solution at fixe potential of 0.4V.


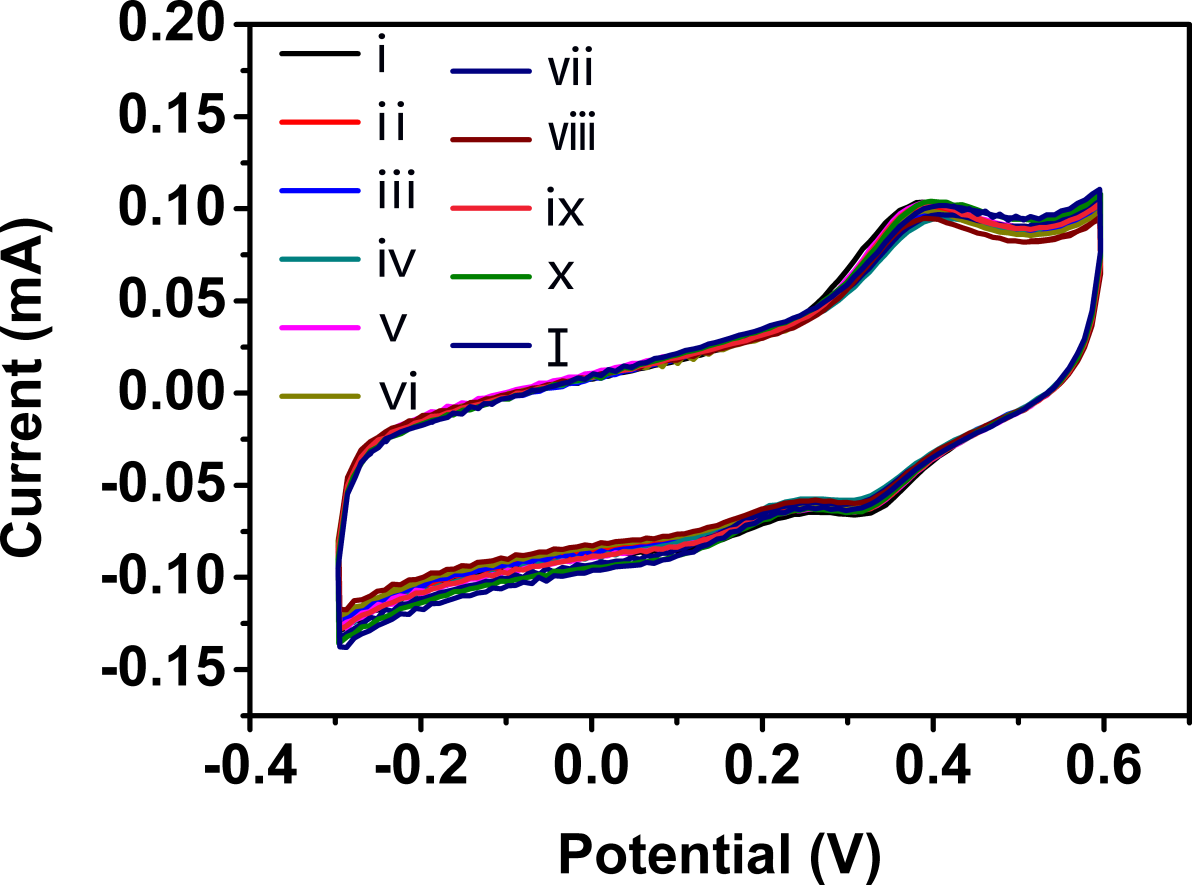


**Fig. S7.** The CV curves of the Pd/NPG wire biosensor in the solution of 100 μΜ DA in the presence of AA, UA, NE, EP and CC (ⅰ: DA (100 mM); ⅱ: DA (100 mM) + AA (0.05 mM); ⅲ DA (100 mM) + AA (0.5 mM); ⅳ: DA (100 mM) + UA (0.3 mM); ⅴ: DA (100 mM) + UA (3 mM); ⅵ: DA (100 mM) + NE (0.1 mM); ⅶ: DA (100 mM) + NE (1 mM); ⅷ: DA (100 mM) + EP (0.6 mM); ⅸ: DA (100 mM) + EP (6 mM); ⅹ: DA (100 mM) +CC (0.5 mM); Ⅰ: DA (100 mM) + CC (5 mM) ).


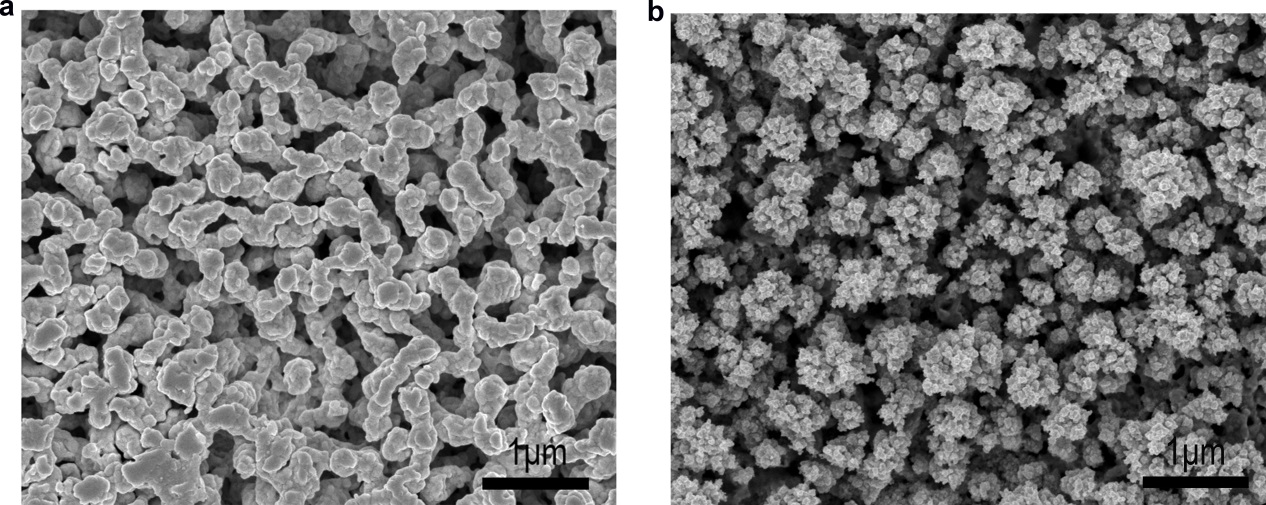


**Fig. S8.** (a) SEM image of NPG wire obtained from the Pd/NPG wire electrode by etching in concentrated HNO_3_. (b) SEM of the reconstructed Pd/NPG wire on this NPG wire.





**Fig. S9.** The CV curves of five parallel sample of Pd/NPG wire biosensor.

**Table S1 The comparison of the detection properties of Pd/NPG wire biosensors with other nonenzymatic electrochemical dopamine biosensors.**

| NP^a^ modified matrix | Method | Linearity (μΜ) | Sensitivity  (μA μΜ^-1^) | Detection limit (μΜ) | Ref. |
| --- | --- | --- | --- | --- | --- |
| ERGO^b^/GCE^c^ | DPV^d^ | 0.5-60 | 0.482 | 0.5 | [^1^](#_ENREF_1) |
| GONR^e^/GCE | DPV | 1-6 | 0.33 | 1 | [^2^](#_ENREF_2) |
| GO^f^/PAN^g^ | Amperometry | 1.0-14 | 0.265 | 0.5 | [^3^](#_ENREF_3) |
| NG^h^ | LSV^i^ | 0.5-170 | 0.032 | 0.25 | [^4^](#_ENREF_4) |
| Tyrosinase/NiO/ITO | CV^j^ | 2-100 | 0.06 | 1.04 | [^5^](#_ENREF_5) |
| MWCNT^k^/GCE | DPV | 1-50 | 2.31 | 1 | [^6^](#_ENREF_6) |
| PUCNT^l^/GCE | DPV | 1-50 | 1.04 | 1 | [^6^](#_ENREF_6) |
| Pd-NPG^m^ wire | CV | 1-220 | 1.19 | 1 | This work |

^a^ nanoparticle, ^b^ electrochemically reduced graphene oxide, ^c^ glassy carbon electrode, ^d^ differential pulse voltammetry, ^e^ graphene oxide nanoribbon, ^f^ graphene oxide, ^g^ polyacrylonitrile, ^h^ N-doped grapheme, ^i^ linear sweep voltammetry, ^j^ cyclic voltammetry, ^k^ multi-walled carbon nanotubes, ^l^ partially unzipped carbon nanotubes, ^m^ nanoporous gold

**Table S2 Selectivity studies of the biosensor in the solution of 100 μΜ DA in the presence of AA and UA**

| **Dopamine and interference** | **anodic peak current**  **(I_pa_; mA)** | **RSD(%)** |
| --- | --- | --- |
| DA (100 $\mu$M) | 0.1037 | 2.93 |
| DA (100 $\mu$M) + AA (0.05 mM) | 0.1017 | 5.24 |
| DA (100 $\mu$M) + AA (0.5 mM) | 0.0976 | 5.43 |
| DA (100 $\mu$M) + UA (0.3 mM) | 0.0949 | 4.85 |
| DA (100 $\mu$M) + UA (3 mM) | 0.1036 | 4.59 |
| DA (100 $\mu$M) + NE (0.1 mM) | 0.0989 | 7.38 |
| DA (100 $\mu$M) + NE (1 mM) | 0.0969 | 6.46 |
| DA (100 $\mu$M) + EP (0.6 mM) | 0.0947 | 6.94 |
| DA (100 $\mu$M) + EP (6 mM) | 0.1026 | 5.93 |
| DA (100 $\mu$M) + CC (0.5 mM) | 0.1041 | 9.46 |
| DA (100 $\mu$M) + CC (5 mM) | 0.1017 | 8.73 |

1. Yang, L., Liu, D., Huang, J.S. & You, T.Y. Simultaneous determination of dopamine, ascorbic acid and uric acid at electrochemically reduced graphene oxide modified electrode. *Sensors and Actuators B-Chemical* **193**, 166-172 (2014).

2. Zhang, X., Gu, S.Q. & Ding, Y.P. Simultaneous detection of roxithromycin and dopamine using a sensor platform based on poly(sulfosalicylic acid) and its application in human serum studies. *Analytical Methods* **6**, 3316-3321 (2014).

3. Bao, Y. et al. Graphene Oxide-Templated Polyaniline Microsheets toward Simultaneous Electrochemical Determination of AA/DA/UA. *Electroanalysis* **23**, 878-884 (2011).

4. Sheng, Z.H. et al. Electrochemical sensor based on nitrogen doped graphene: Simultaneous determination of ascorbic acid, dopamine and uric acid. *Biosensors & Bioelectronics* **34**, 125-131 (2012).

5. Roychoudhury, A., Basu, S. & Jha, S.K. Dopamine biosensor based on surface functionalized nanostructured nickel oxide platform. *Biosensors & Bioelectronics* **84**, 72-81 (2016).

6. Hu, H.F., Song, Y.P., Feng, M. & Zhan, H.B. Carbon nanomaterials for simultaneous determination of dopamine and uric acid in the presence of ascorbic acid: from one-dimensional to the quasi one-dimensional. *Electrochimica Acta* **190**, 40-48 (2016).
